# Ageist Policies That Favor Older People: What Do Older People Think?

**DOI:** 10.1093/geroni/igab046.517

**Published:** 2021-12-17

**Authors:** Hakan Jonson, Tove Harnett

**Affiliations:** 1 Lund University, Lund University, Skane Lan, Sweden; 2 Lund University, Lund, Skane Lan, Sweden

## Abstract

Policies on supportive services have frequently used chronological age to determine rights and needs of people within the adult population. Such policies have been described as ageist, but could also be regarded as favoring older people in cases where chronological age is used as a proxy for needs. In Sweden, municipalities have recently been allowed to grant people above a certain age some home care services without individual needs testing, and several political parties have suggested that a nursing home guarantee at the age of 85 should be introduced. The aim of the study that this presentation reports on was to investigate views among older people on age as an organizing principle for distributing eldercare services. Data was collected through an online surveys to members of pensioners’ organisations (N=1540). Respondents were asked about their views on a number of age-related policies that are used or proposed as part of the eldercare system in Sweden. The analysis revealed a general support for the use of chronological age as a proxy for needs. This suggest that respondents used an interest groups perspective and supported stereotypical arrangements that favored older people. When free-text answers were included in the analysis, it became evident that the use of chronological age was not related to the problem of ageism. In the presentation we will discuss the potential gap between anti-ageism and views of older people and what a framework on ageism brings into the moral economy of eldercare.

